# The Predictive Competing Endogenous RNA Regulatory Networks and Potential Prognostic and Immunological Roles of Cyclin A2 in Pan-Cancer Analysis

**DOI:** 10.3389/fmolb.2022.809509

**Published:** 2022-04-11

**Authors:** Shenyong Chen, Zhijia Zhao, Xiaobo Wang, Qi Zhang, Li Lyu, Bo Tang

**Affiliations:** ^1^ Department of Pathology, The Second Affiliated Hospital of Dalian Medical University, Dalian, China; ^2^ Department of Hematology, The Second Affiliated Hospital of Dalian Medical University, Dalian, China

**Keywords:** CCNA2, pan-cancer analysis, ceRNA network, survival analysis, immune infiltration

## Abstract

Although accumulating evidence has verified the relationship between CCNA2 and cancers, no pan-cancer analysis about the function and the upstream molecular mechanism of CCNA2 is available. For the first time, we analyzed potential oncogenic roles of CCNA2 in 33 cancer types *via* The Cancer Genome Atlas (TCGA) database. Overexpression of CCNA2 is widespread in almost all cancer types, and it is related to poor prognosis and advanced pathological stages in most cases. Moreover, we conducted upstream miRNAs and lncRNAs of CCNA2 to establish upstream regulatory networks in kidney renal clear cell carcinoma (LINC00997/miR-27b-3p/CCNA2), liver hepatocellular carcinoma (SNHG16, GUSBP11, FGD5-AS1, LINC00630, CD27-AS1, LINC00997/miR-22-3p/CCNA2, miR-29b-3p/CCNA2, miR-29c-3p/CCNA2, and miR-204-5p/CCNA2), and lung adenocarcinoma (miRNA-218-5p/CCNA2 and miR-204-5p/CCNA2) by expression analysis, survival analysis, and correlation analysis. The CCNA2 expression is positively correlated with Th2 cell infiltration and negatively correlated with CD4^+^ central memory and effector memory T-cell infiltration in different cancer types. Furthermore, CCNA2 is positively associated with expressions of immune checkpoints (CD274, CTLA4, HAVCR2, LAG3, PDCD1, and TIGIT) in most cancer types. Our first CCNA2 pan-cancer study contributes to understanding the prognostic and immunological roles and potential upstream molecular mechanisms of CCNA2 in different cancers.

## Introduction

Cyclins are established regulators of the cell cycle through activation of a specific family of kinases, the cyclin-dependent kinases (CDKs). Cyclin A2 (CCNA2) is a member of the cyclin family that, promotes transition through G1/S and G2/M by binding and activating cyclin-dependent kinase 1 (CDK1) and cyclin-dependent kinase 2 (CDK2) (Baumann, 2016). In addition, there are additional functions of CCNA2, such as cytoskeleton dynamic and cell motility (Bendris et al., 2012). CCNA2 overexpression has been reported in certain types of cancer, such as pancreatic ductal adenocarcinoma (Dong et al., 2019), stomach adenocarcinoma (Zhang et al., 2018a), and hepatocellular carcinoma (Zhang et al., 2021). Moreover, its overexpression indicates poor prognosis of patients in some cancer types (Ko et al., 2013; Zhang et al., 2018a; Dong et al., 2019; Zhang et al., 2021). CCNA2 has been identified as a tumor therapeutic target involved in the processes of cell proliferation (Peng et al., 2018; Wang et al., 2021a; Xiong et al., 2021). However, there is still no pan-cancer evidence for CCNA2 in various tumor types based on large clinical data. Recently, large numbers of functional genomics databases have become available on public platforms. The Cancer Genome Atlas (TCGA) project has generated genomic, epigenomic, transcriptomic, and proteomic data on 33 different cancer types. In addition, TCGA has clinical data such as prognosis and pathological stage. Therefore, we conducted a pan-cancer analysis of CCNA2 using the TCGA project.

Noncoding RNAs (ncRNAs) lack protein-encoding capabilities. MicroRNAs (miRNAs) are a family of ncRNAs with a length of 20–25 nucleotides, which widely participate in post-transcriptional regulation by binding to complementary sites within the 3′-untranslated region (3′-UTR) of target mRNAs (Tang et al., 2018). LncRNAs are defined as a subtype of ncRNA longer than 200 nucleotides. The competing endogenous RNA (ceRNA) hypothesis described that lncRNAs, mRNAs, and transcribed pseudogenes compete for binding miRNAs *via* miRNA response elements (MREs) (Salmena et al., 2011). In other words, when lncRNAs competitively sequester miRNAs from their targeted mRNAs through complementary base pairing, known as “sponging”, they increase the mRNA expression by decreasing the binding of miRNAs and mRNAs (Karreth and Pandolfi, 2013). Accumulating evidence has shown that lncRNAs play a critical role in tumor development and metastasis by acting as a ceRNA for miRNAs (Wang et al., 2017; Yang et al., 2018; Qiao et al., 2019). Moreover, several lncRNAs have been verified as ceRNAs of CCNA2 in tumors. DNAH17-AS1 promotes the development of non-small cell lung cancer by targeting the miR-877-5p/CCNA2 pathway (Du et al., 2020). Data from the Gene Expression Omnibus (GEO) database shows that LINC00665 promotes lung adenocarcinoma by miR-let-7b-CCNA2 (Du et al., 2020). HMGA1P4 regulates CCNA2 by miR‐301b/miR‐508 in gastric cancer (Zhang et al., 2019). However, the miR‐301b/miR‐508 expression was not verified to be inversely correlated with CCNA2 mRNA and HMGA1P4.

Immune cells and inflammatory cytokines in the tumor microenvironment could influence tumors’ development and occurrence, and immune escape plays a key role in the process of tumorigenesis and development (Elinav et al., 2013). Tumor cells inhibit the activation of T cells to avoid antitumor immune attacks through immune checkpoints. Therefore, targeting immune checkpoints to remove inhibitory effects is a notable method of tumor immunotherapy (Tsai and Hsu, 2017). Cluster of differentiation 274 (CD274/PD-L1), programmed cell death protein 1 (PDCD1/PD-1), and cytotoxic T lymphocyte-associated antigen-4 (CTLA-4/CD152) are frequently clinically targeted immune checkpoints in recent years. However, immunotherapy targeting the above immune checkpoints is ineffective in many tumor patients. Therefore, more immune checkpoints are needed to expand the therapeutic range. Therapies targeting novel immune checkpoints such as lymphocyte activation gene-3 (LAG-3/CD223), hepatitis A virus cellular receptor 2 (HAVCR2/TIM-3), and T-cell immunoreceptor with Ig and ITIM domains (TIGIT) have been under clinical trials (Sasidharan Nair and Elkord, 2018). LAG-3 is upregulated on activated CD4^+^ T cells, CD8^+^ T cells, and a subset of natural killer (NK) cells, and inhibits T-cell responses, cooperating with PD-1 (Anderson et al., 2016). HAVCR2 marks the exhausted or dysfunctional CD8^+^ T cells (Anderson et al., 2016) and has a non-redundant synergy with PD-1 in the suppression of effector T-cell activity (Tsai and Hsu, 2017). TIGIT is confirmed to inhibit the responses of effector T cells in tumors synergizing with PD-1 or HAVCR2 (Tsai and Hsu, 2017; Solomon and Garrido-Laguna, 2018).

For the first time, our research uses the TCGA project to investigate a pan-cancer analysis of CCNA2, including gene expression, clinical survival prognosis, pathological stage, immune cell infiltration, immune cell markers, and immune checkpoints. Moreover, we identified upstream miRNAs and lncRNAs of CCNA2 to establish several ceRNA networks or miRNA-CCNA2 mRNA regulatory networks in kidney renal clear cell carcinoma (KIRC), liver hepatocellular carcinoma (LIHC), and lung adenocarcinoma (LUAD) by expression analysis, survival analysis, and correlation analysis. We believe that our work could assist in predicting the prognosis of patients with many cancer types and deepen our understanding of the potential regulation mechanism of CCNA2.

## Methods

### Data Source and Processing

The Cancer Genome Atlas (TCGA; http://cancergenome.nih.gov), which is a landmark cancer genomics program, has characterized over 20,000 primary cancer and normal samples in 33 cancer types until Sep 2021. Using UCSC Xena (https://xenabrowser.net/), we collected gene expression RNAseq data and survival data from various cancer and normal samples in the TCGA database (Goldman et al., 2020). Genotype-tissue expression (GTEx; http://commonfund.nih.gov/GTEx/) is a gene expression data from 54 normal tissue sites across nearly 1,000 people by RNA sequencing. We used normal samples from GTEx when there were none or <5 normal samples from TCGA in specific cancer types, to compare the CCNA2 expression from cancer and normal tissue.

### CCNA2 Expression Profiles

Perl script and the R language were used to integrate and analyze the data. A Perl script was used to convert “Ensembl_ID” of the transcriptome data that was downloaded from UCSC Xena to the name of the corresponding gene. Then, we chose the “limma” package of the R language to perform difference analysis of the CCNA2 expression in cancer types with five or more adjacent normal tissues from the TCGA project. We took |log2FC|> 1 and an adjusted *p*-value < 0.05 as the cut-off criterion for further CCNA2-ceRNA network analysis. For certain tumor types without normal or <5 normal tissues from the TCGA project, we used the “Expression on Box Plots” module of the GEPIA (Gene Expression Profiling Interactive Analysis) web server (http://gepia.cancer-pku.cn/) to obtain the expression of CCNA2 in these tumor tissues and the normal tissues of the GTEx and TCGA databases (Tang et al., 2017).

### Survival Analysis

We first analyzed overall survival (OS) and disease-free survival (DFS) based on the CCNA2 expression (50% high-expression group and 50% low-expression group) in all cancer types by the “Survival Plots” module of the GEPIA web server. GEPIA uses the Log-rank test, also known as the Mantel–Cox test, for hypothesis testing. To ensure that CCNA2 has a definite OS significance in some tumor types, we further performed OS analysis using the “survival” package in the R language by the Kaplan-Meier survival curve and the log-rank test based on the CCNA2 expression (50% high-expression group and 50% low-expression group) in cancer types with five or more TCGA adjacent normal tissues, and only tumor types with statistical significance by two methods will undergo further CCNA2-ceRNA network analysis. We also performed survival analysis by using the Kaplan–Meier survival curve based on miRNA and lncRNA expressions (at the best cutoff, there was the most significant difference in OS between the high- and the low-expression groups), which used “survival” and “survminer” packages in the R language.

### Prediction of Upstream miRNA/lncRNA

The starBase database (https://starbase.sysu.edu.cn/) was employed to predict miRNA–lncRNA interactions and miRNA–mRNA interactions (Li et al., 2014), and the results were supported by Ago CLIP-seq data. Interactions of miRNA–mRNA were predicted by at least two programs from PITA, RNA22, miRmap, DIANA-microT, miRanda, PicTar, and TargetScan. The interactions of miRNA with lncRNA were predicted by using the miRanda program. According to the mechanism of ceRNA, CCNA2-related ceRNAs were selected according to the following criteria (Zhou et al., 2014; Xu et al., 2015): 1) The CCNA2 (upregulated in tumor, |log2FC|> 1), CCNA2-bound miRNAs (downregulated in tumor) and lncRNAs (upregulated in tumor) were significantly differentially expressed in tumor samples compared with normal samples from TCGA. 2) The lncRNAs and CCNA2 were positively correlated (rho value > 0.2, *p* value < 0.05, Spearman’s rank correlation test) in tumor samples, which were identified as co-expressed lncRNA–mRNA pairs. 3) For a given co-expressed lncRNA–CCNA2 mRNA pair, both CCNA2 and lncRNA in this pair were targeted and co-expressed negatively with a certain miRNA (rho value < −0.2, *p* value < 0.05, Spearman’s rank correlation test) in tumor samples. ceRNA networks and miRNA-CCNA2 networks were presented using the Cytoscape web tool (http://js.cytoscape.org). All analyses were performed in R 4.1.0 (https://www.r-project.org/).

### Immune Infiltration

TIMER2.0 (http://timer.cistrome.org/) web server is a comprehensive resource for systematical analysis of immune infiltrates across diverse cancer types (Li et al., 2017; Li et al., 2020a). We used it to explore the association between CCNA2 expression and immune infiltrates (B cells, CD4+T cells, CD8+T cells, myeloid dendritic cells, macrophages, monocytes, NK cells, Tregs, and neutrophils) across TCGA tumors. Moreover, we used the “gene correlation” module of TIMER2.0 to explore the correlations between CCNA2 and expressions of immune checkpoints (CD274, CTLA4, HAVCR2, LAG3, PDCD1, and TIGIT). The *p*-values and partial rho values were obtained *via* the purity-adjusted Spearman’s rank correlation test.

## Results

### CCNA2 Is Overexpressed in Most Cancers

**FIGURE 1:**
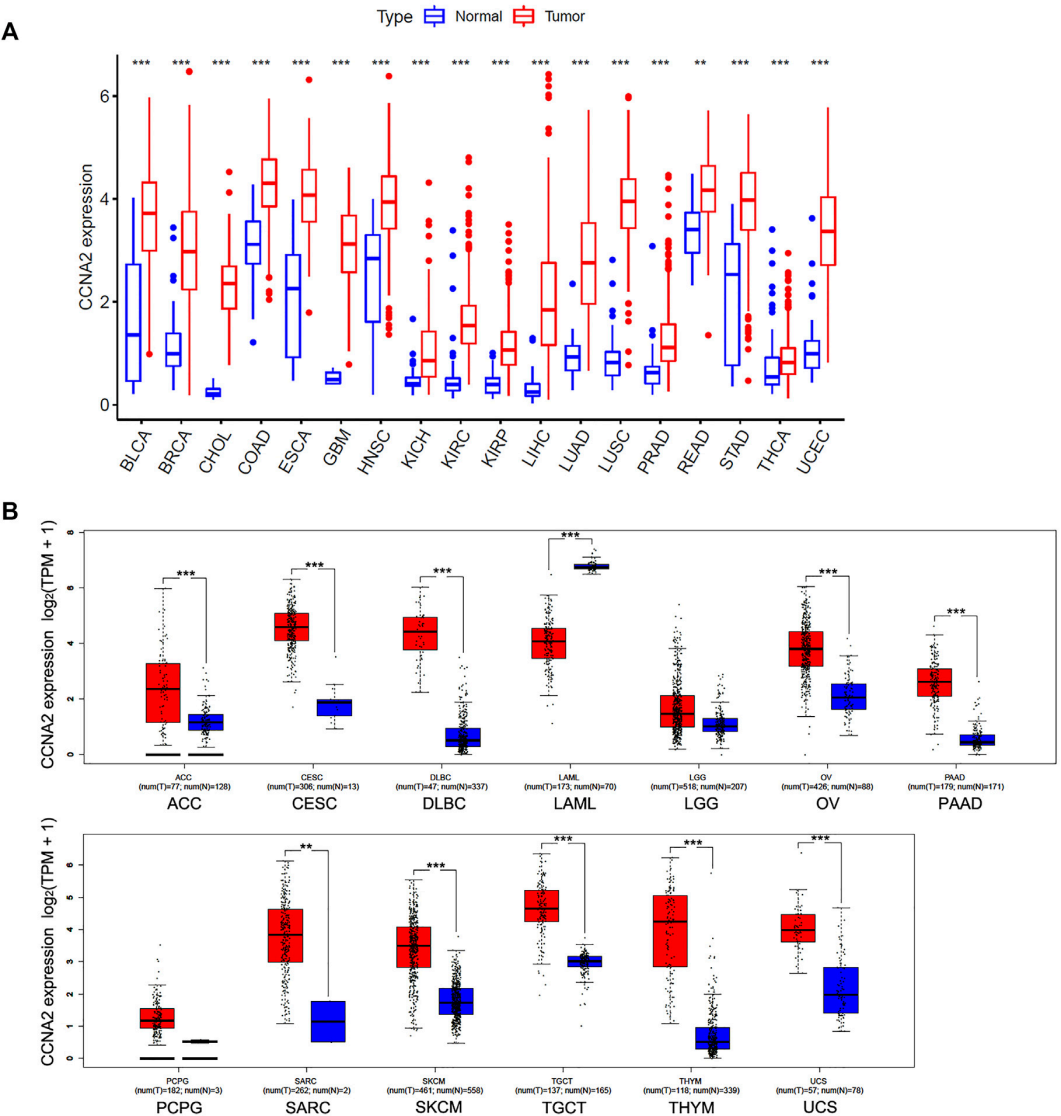
Expression Analysis of CCNA2 in various cancers. **(A)** The expression of the CCNA2 in 18 cancer types with five or more normal tissues from the TCGA project *via* R language. **(B)** The expression of the CCNA2 in 13 other cancer types, normal tissues from GTEx project, or TCGA + GTEx as controls. **p < 0.01, ***p < 0.001.

We first analyzed the CCNA2 expression in cancer types with five or more adjacent normal tissues from the TCGA project. As graphed in Figure 1A, the expression level of CCNA2 in all 18 cancers of bladder urothelial carcinoma (BLCA), breast invasive carcinoma (BRCA), cholangiocarcinoma (CHOL), colon adenocarcinoma (COAD), esophageal carcinoma (ESCA), glioblastoma multiforme (GBM), head and neck squamous cell carcinoma (HNSC), kidney chromophobe (KICH), KIRC, kidney renal papillary cell carcinoma (KIRP), LIHC, LUAD, lung squamous cell carcinoma (LUSC), prostate adenocarcinoma (PRAD), rectum adenocarcinoma (READ), stomach adenocarcinoma (STAD), thyroid carcinoma (THCA), and uterine corpus endometrial carcinoma (UCEC) is higher than their adjacent normal tissues (*p* < 0.01 in READ, and *p* < 0.001 in other cancer types). After including the normal tissues from the GTEx project as controls, we determined the expression level of CCNA2 in other cancer types. As is shown in Figure 1B, the expression of CCNA2 mRNA is higher in adrenocortical carcinoma (ACC), cervical squamous cell carcinoma and endocervical adenocarcinoma (CESC), diffuse large B-cell lymphoma (DLBC), ovarian serous cystadenocarcinoma (OV), pancreatic adenocarcinoma (PAAD), sarcoma (SARC), skin cutaneous melanoma (SKCM), testicular germ cell tumors (TGCT), thymoma (THYM), and uterine carcinosarcoma (UCS, *p* < 0.01 in SARC, and *p* < 0.001 in other cancer types). However, CCNA2 mRNA is lower in acute myeloid leukemia (LAML) than that in normal tissues from the GTEx project (*p* < 0.001), and we do not obtain a significant difference in brain lower grade glioma (LGG) and pheochromocytoma and paraganglioma (PCPG).

### Significant Prognostic Value of CCNA2 in Cancers

**FIGURE 2:**
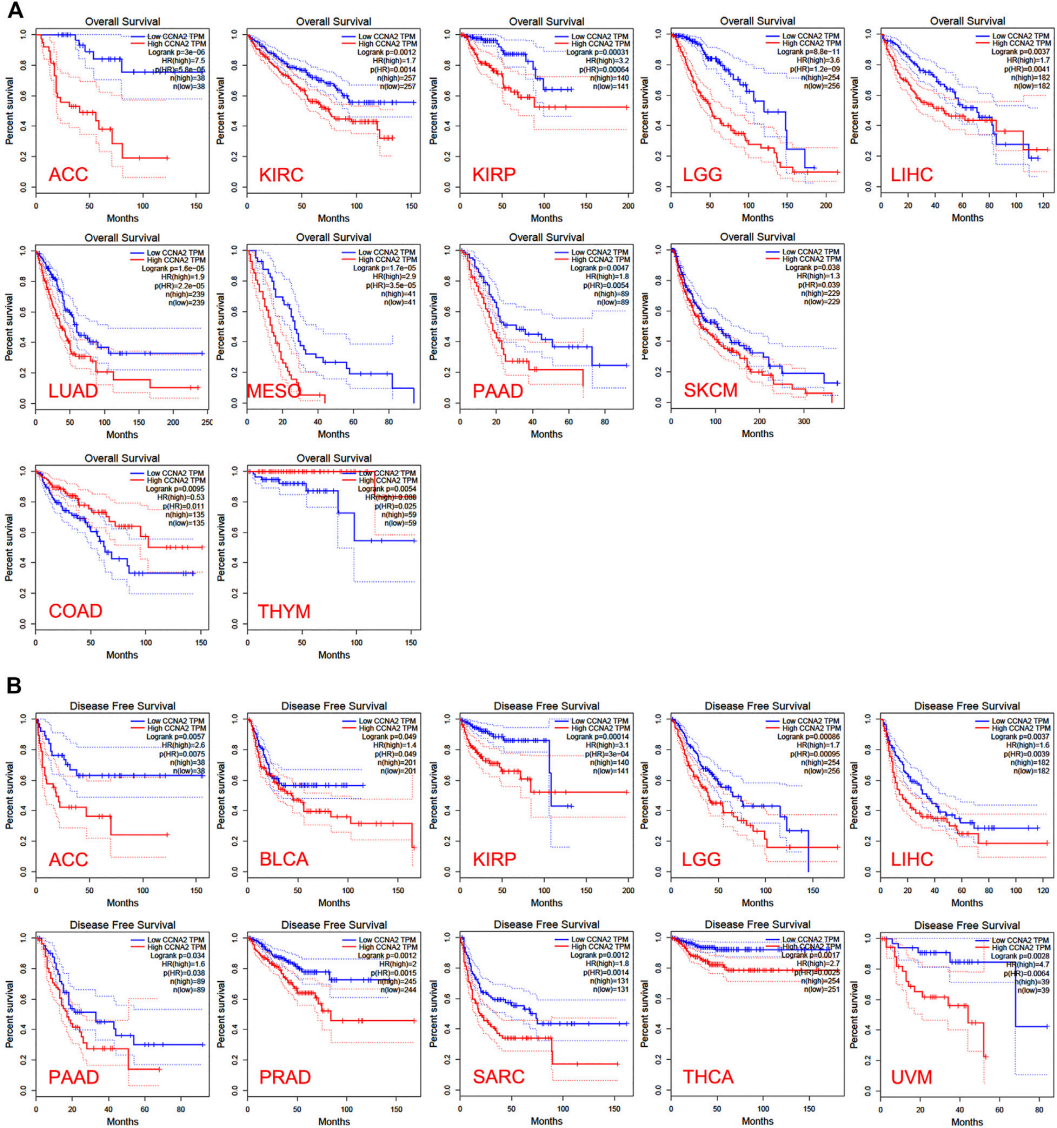
Overall survival analysis **(A)** and disease-free survival analysis **(B)** based on the CCNA2 expression in various cancers.

**FIGURE 3:**
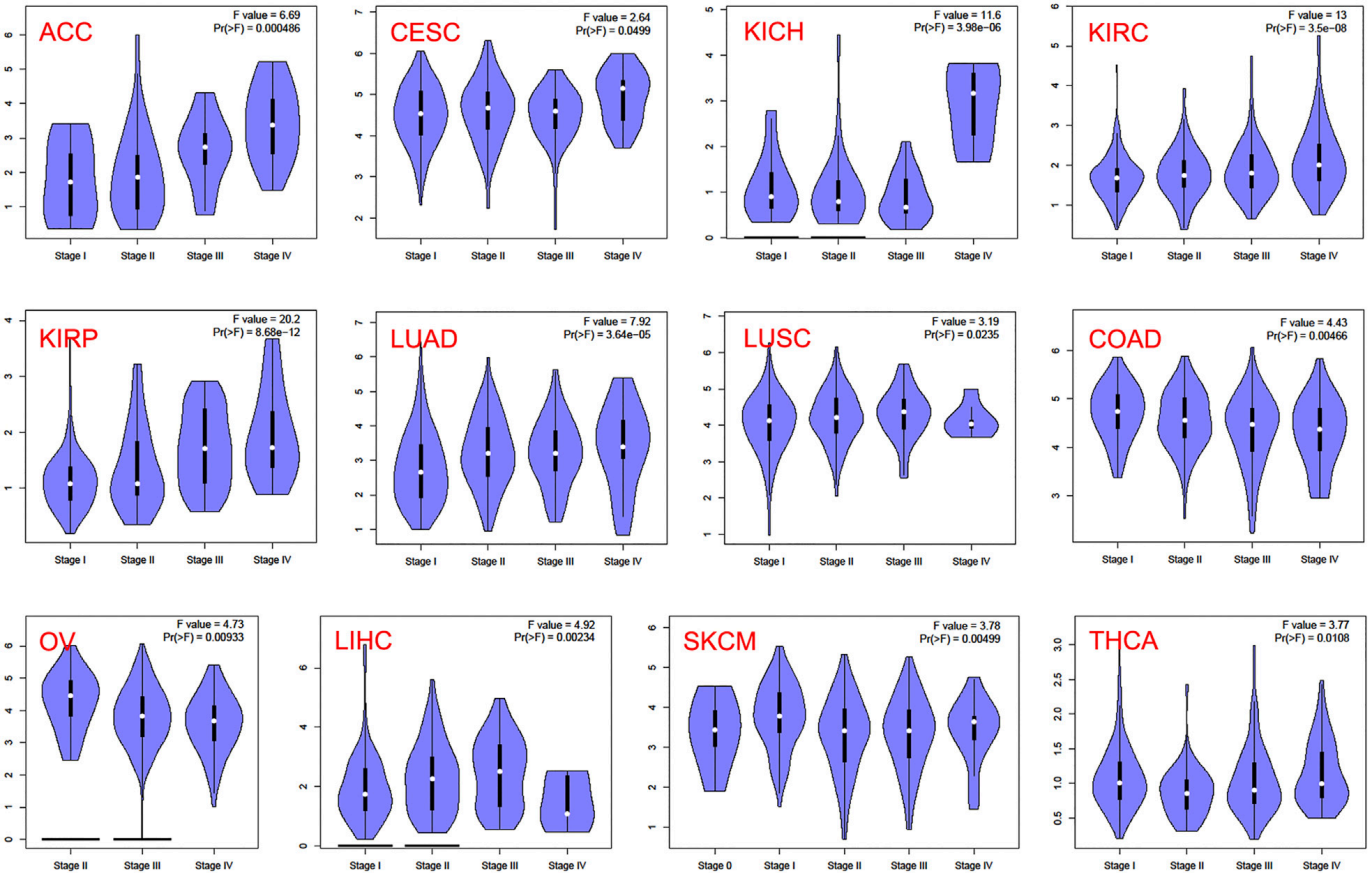
CCNA2 expression of various pathological stages in various cancers.

In the TCGA project, we used the “Survival Plots” module of GEPIA to analyze the relationship between CCNA2 expression level and the clinical prognosis of patients with different cancer types. We separated the cancer cases into high-expression groups (50%) and low-expression groups (50%) according to the expression levels of CCNA2. As is shown in Figure 2A, the high expression of CCNA2 is related to poor prognosis of overall survival (OS) in cancers of ACC (*p <* 0.001), KIRC (*p* = 0.0014), KIRP (*p* < 0.001), LGG (*p* < 0.001), LIHC (*p* = 0.0041), LUAD (*p* < 0.001), MESO (*p* < 0.001), PAAD (*p* = 0.0047), and SCKM (*p* = 0.011, Figure 1A). In addition, PRAD nearly reaches statistical significance (*p* = 0.054). However, high expression of CCNA2 is related to better OS in COAD (*p* = 0.039) and THYM (*p* = 0.025, Figure 2A). Disease-free survival (DFS) data indicates high expression of CCNA2 is related to poor prognosis in cancers of ACC (*p* = 0.0057), BLCA (*p* = 0.049), KIRP (*p* < 0.001), LGG (*p* < 0.001), LIHC (*p* = 0.0039), PAAD (*p* = 0.038), PRAD (*p* = 0.0015), SARC (*p* = 0.0014), THCA (*p* = 0.0025), UVM (*p* = 0.0064) (Figure 2B). In addition, KICH (*p* = 0.054), KIRC (*p* = 0.06), and SKCM (*p* = 0.061) nearly reach statistical significance. We also employed the “Pathological Stage Plot” module of GEPIA2 to observe the correlation between CCNA2 expression and the pathological stages of cancers. As is shown in Figure 3, high expression of CCNA2 is generally related to advanced staging in ACC, CESC, KICH, KIRC, KIRP, LUAD, and LUSC, and it is related to better staging in COAD and OV (all *p* values *<* 0.05). The results are also statistically significant in LIHC, SKCM, and THCA but do not have a tendency (all *p* values *<* 0.05).

### Predictive Analysis of Upstream miRNA of CCNA2

**FIGURE 4:**
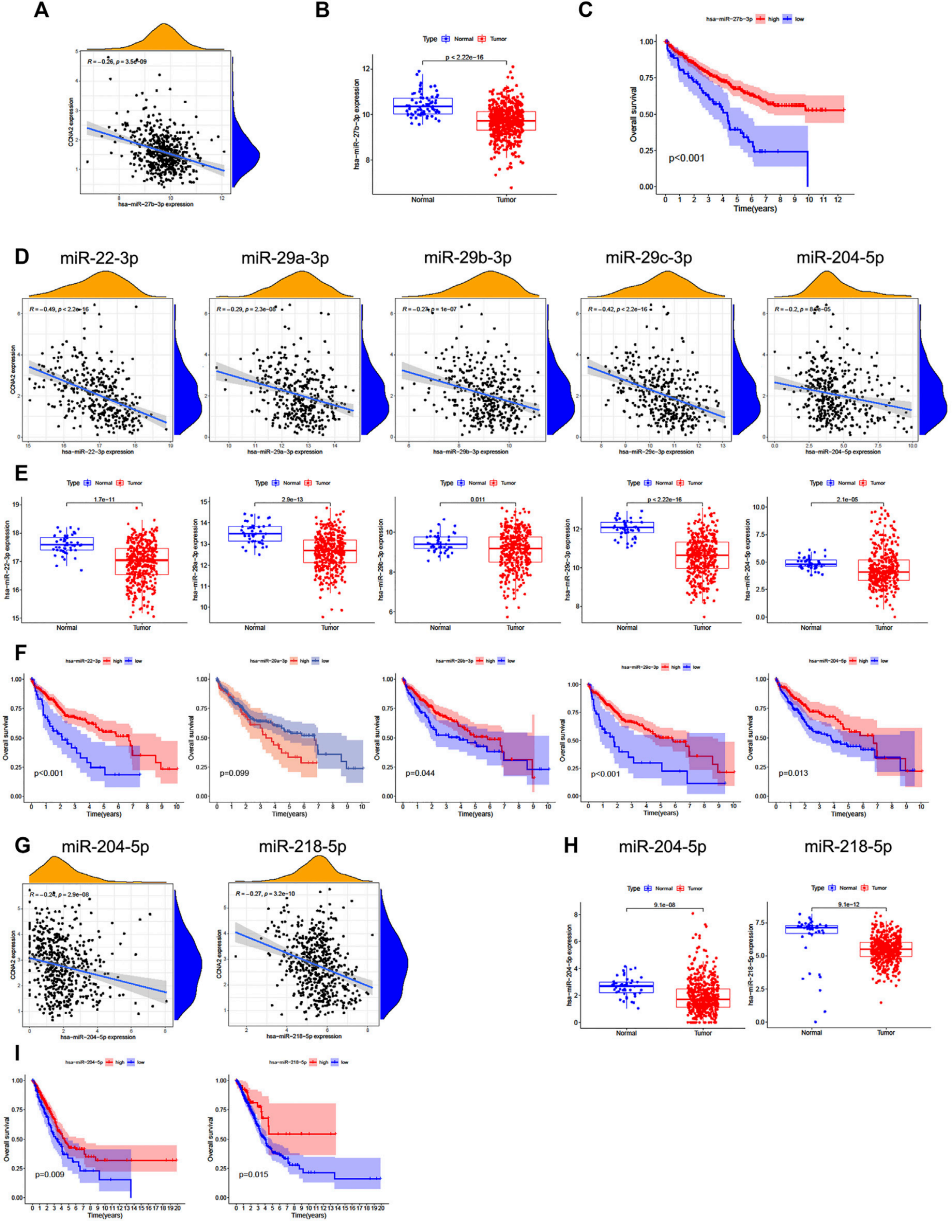
Predictive analysis of upstream miRNA of CCNA2. MicroRNA expression was shown as log2 (miRNA+1). **(A)** The negative correlation between miR-27b-3p and CCNA2 in KIRC. **(B)** The expression of miR-27b-3p in KIRC and normal tissues (num (T) = 545; num (N) = 71). **(C)** Overall survival analysis based on the miR-27b-3p expression in KIRC. **(D)** The negative correlation between miRNAs (miR-22-3p, miR-29c-3p, miR-29a-3p, miR-29b-3p, and miR-204-5p) and CCNA2 in LIHC. **(E)** The expression of miRNAs (miR-22-3p, miR-29c-3p, miR-29a-3p, miR-29b-3p, and miR-204-5p) in LIHC and normal tissues (num (T) = 375; num (N) = 50). **(F)** Overall survival analysis based on expression of miRNAs (miR-22-3p, miR-29c-3p, miR-29a-3p, miR-29b-3p, and miR-204-5p) in LIHC. **(G)** The negative correlation between miRNAs (miR-204-5p and miR-218-5p) and CCNA2 in LUAD. **(H)** The expression of miRNAs (miR-204-5p and miR-218-5p) in LUAD and normal tissues (num (T) = 521; num (N) = 46). **(I)** Overall survival analysis based on the expression of miRNAs (miR-204-5p and miR-218-5p) in LUAD.

We further analyzed the predictive ceRNA network in cancer types with enough adjacent normal tissues of TCGA project. We chose the cancer types in which high CCNA2 expression is associated with poor OS by the Kaplan–Meier survival curve and log-rank test simultaneously (Table 1). The starBase database was used for predicting upstream miRNAs of CCNA2, and these 52 CCNA2-bound miRNAs have been supported by the CLIP-Seq experiment (Table 2). MicroRNA-27b-3p expression is significantly negatively correlated with CCNA2 in KIRC (R = −0.26, *p* < 0.001, Figure 4A), and it is lower in KIRC tissues than in normal tissues (*p* < 0.001, Figure 4B). KIRC patients with decreased miR-27b-3p expression have a poor prognosis (*p* < 0.001, Figure 4C). The expression of miR-22-3p (R = -0.49), miR-29c-3p (R = −0.42), miR-29a-3p (R = −0.29), miR-29b-3p (R = −0.29) and miR-204-5p (R = −0.20) is negatively correlated with CCNA2 in LIHC (all *p* values < 0.001, Figure 4D), and their expression is lower in LIHC tissues than in normal tissues (miR-29b-3p *P* = 0.011, other *p* values < 0.001, Figure 4E). LIHC patients with decreased expression levels of miR-22-3p (*p* < 0.001), miR-29b-3p (*p* = 0.044), miR-29c-3p (*p* < 0.001), miR-204-5p (*p* = 0.013) are correlated with poor prognosis (Figure 4F). However, miR-29a-3p shows the opposite tendency. In LUAD, there are ten miRNAs significantly negatively correlated with CCNA2 (all *p* values < 0.001, miR-204-5p and miR-218-5p are shown in Figure 4G, other miRNAs are shown in Supplementary Figure S1). However, only the expressions of miR-204-5p and miR-218-5p are lower in LUAD tissues than in normal tissues (*p* < 0.001, Figure 4H). LUAD patients with decreased expression levels of miR-204-5p (*p* = 0.009) and miR-218-5p (*p* = 0.015) are correlated with poor OS prognosis (Figure 4I).

**TABLE 1:** CCNA2 associated with poor OS by the Kaplan–Meier survival curve and log-rank test.

	KM	HR	HR.95L	HR.95H	Cox *p* value
KIRC	6.63E-05	2.1874264	1.764223	2.712149	9.69E-13
LIHC	0.0123095	1.2985597	1.135964	1.484428	0.0001293
LUAD	9.16E-05	1.304526	1.143175	1.488651	7.94E-05

**TABLE 2:** Predicting upstream miRNAs of CCNA2, and these miRNAs have been supported by the CLIP-Seq experiment.

hsa-miR-19a-3p	hsa-miR-211-5p	hsa-miR-29c-3p	hsa-miR-499a-5p	hsa-miR-1323
hsa-miR-19b-3p	hsa-miR-218-5p	hsa-miR-301a-3p	hsa-miR-513a-5p	hsa-miR-548o-3p
hsa-miR-22-3p	hsa-miR-219a-5p	hsa-miR-130b-3p	hsa-miR-508-3p	hsa-miR-4295
hsa-miR-27a-3p	hsa-miR-200b-3p	hsa-miR-381-3p	hsa-miR-588	hsa-miR-3666
hsa-miR-29a-3p	hsa-miR-27b-3p	hsa-miR-148b-3p	hsa-miR-641	hsa-miR-4662a-5p
hsa-miR-29b-3p	hsa-miR-130a-3p	hsa-miR-448	hsa-miR-454-3p	hsa-miR-4701-5p
hsa-miR-199a-5p	hsa-miR-145-5p	hsa-miR-429	hsa-miR-340-5p	hsa-miR-4782-3p
hsa-miR-208a-3p	hsa-miR-152-3p	hsa-miR-410-3p	hsa-miR-300	hsa-miR-892c-5p
hsa-miR-148a-3p	hsa-miR-150-5p	hsa-miR-495-3p	hsa-miR-877-5p	
hsa-miR-199b-5p	hsa-miR-188-5p	hsa-miR-524-5p	hsa-miR-301b-3p	
hsa-miR-204-5p	hsa-miR-200c-3p	hsa-miR-520d-5p	hsa-miR-208b-3p	

### Predictive Analysis of Upstream lncRNAs of CCNA2-Bound miRNAs

**FIGURE 5:**
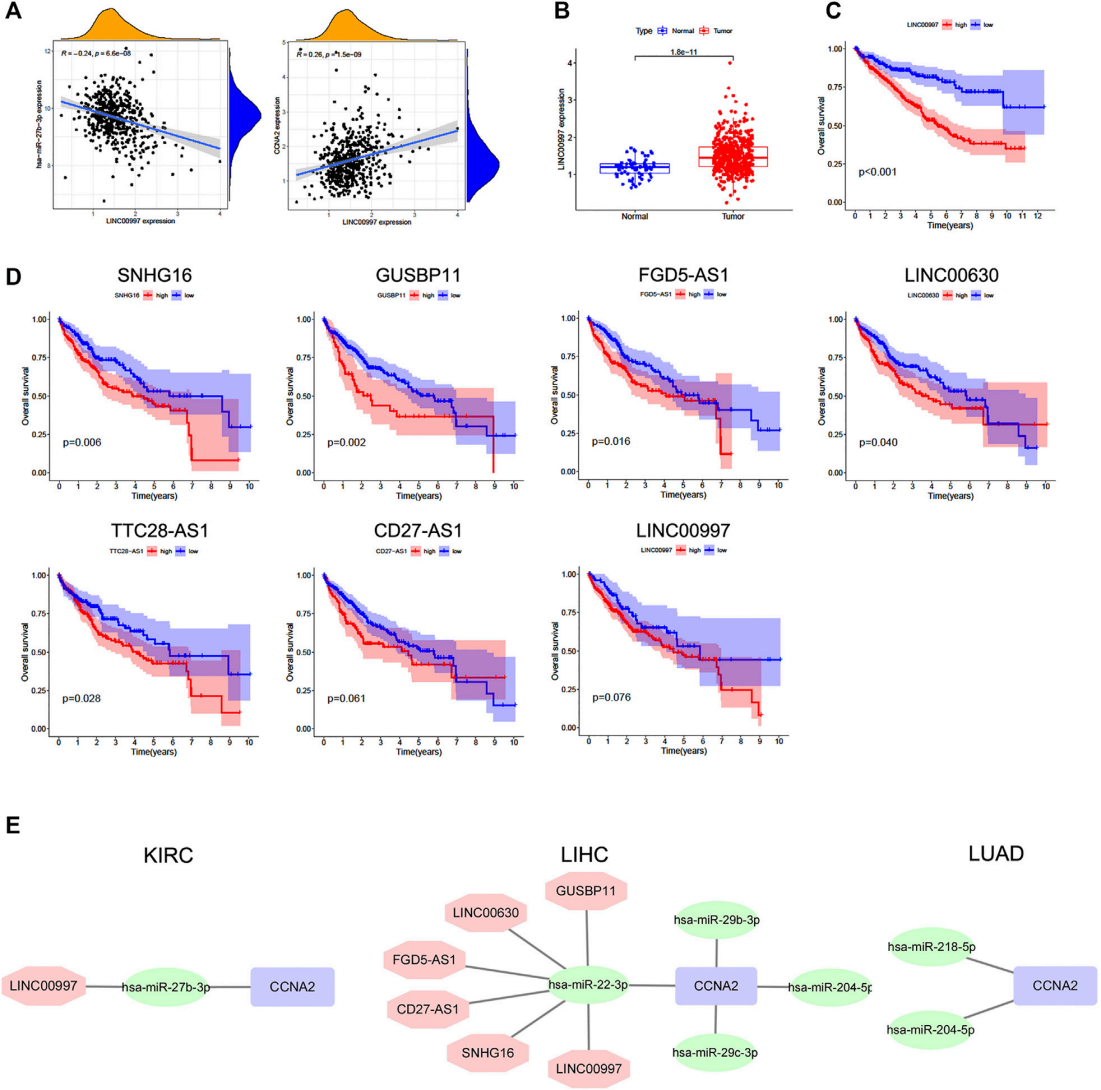
Predictive analysis of upstream lncRNAs of CCNA2-bound miRNAs. The lncRNA expression was shown as log2 (miRNA+1). **(A)** Correlation of LINC00997 expression with miR-27b-3p (left) and CCNA2 (right) in KIRC, num = 545. **(B)** The expression of LINC00997 in KIRC and normal tissues, (num (T) = 545; num (N) = 71). **(C)** Overall survival analysis based on the LINC00997 expression in KIRC, num = 545. **(D)** Overall survival analysis based on miR-22-3p-bound lncRNAs (SNHG16, GUSBP11, FGD5-AS1, TLINC00630, TTC28-AS1, CD27-AS1, and LINC00997) expression in LIHC, num = 375. **(E)** Predictive upstream regulatory networks of CCNA2 in KIRC, LIHC, and LUAD.

The starBase database was used to predict upstream lncRNAs for the above-mentioned miRNAs with expression and survival significance in KIRC, LIHC, and LUAD. According to the ceRNA mechanism, lncRNAs should be positively correlated with CCNA2 mRNA and negatively correlated with miRNAs, and the expression of lncRNAs should be higher than in normal tissues. In KIRC, LINC00997 is significantly negatively correlated with miR-27b-3p (R = −0.24, *p* < 0.001), and it is positively correlated with CCNA2 (R = 0.26, *p* < 0.001, Figure 5A). The expression of LINC00997 is higher in KIRC tissues than in normal tissues (*p* < 0.001, Figure 5B). In addition, KIRC patients with elevated LINC00997 expression levels show poor prognosis (*p* < 0.001, Figure 5C). In LIHC, SNHG16 (R = -0.40), GUSBP11 (R = −0.37), FGD5-AS1 (R = −0.32), TTC28-AS1 (R = −0.29), LINC00630 (R = −0.28), CD27-AS1 (R = −0.25), LINC02381 (R = -0.24), H19 (R = −0.22) and LINC00997 (R = −0.22) are significantly negatively correlated with miR-22-3p (all *p* values < 0.001, Table 3), while only 7 lncRNAs of them are higher in LIHC tissues compared to normal tissues (all *p* values < 0.001, Table 3). The expression of SNHG16 (R = 0.30), GUSBP11 (R = 0.31), FGD5-AS1 (R = 0.43), TLINC00630 (R = 0.43), CD27-AS1 (R = 0.28) and LINC00997 (R = 0.22) is positively correlated with CCNA2 in LIHC (all *p* values < 0.001, Table 4). Besides, TTC28-AS1 and CCNA2 in LIHC nearly reach a positive correlation (R = 0.196, Table 4). Furthermore, increased expression of SNHG16 (*p* = 0.006), GUSBP11 (*p* = 0.002), FGD5-AS1 (*p* = 0.016), TLINC00630 (*p* = 0.04) and TTC28-AS1 (*p* = 0.028) is significantly related to poor OS in LIHC, and CD27-AS1 (*p* = 0.061) and LINC00997 (*p* = 0.076) nearly reach significance in OS (Figure 5D). There is no lncRNA negatively correlated with other CCNA2-bound miRNAs (miR-29b-3p, miR-29c-3p, and miR-204-5p) in LIHC. Similar results were found in LUAD (miR-218-5p and miR-204-5p). Taken together, we summarized LINC00997/miR-27b-3p/CCNA2 ceRNA network in KIRC; SNHG16, GUSBP11, FGD5-AS1, LINC00630, CD27-AS1 and LINC00997/miR-22-3p/CCNA2 ceRNA network, miR-29b-3p/CCNA2, miR-29c-3p/CCNA2, and miR-204-5p/CCNA2 networks in LIHC; and miR-218-5p/CCNA2 and miR-204-5p/CCNA2 networks in LUAD (Figure 5E).

**TABLE 3:** lncRNAs are significantly negatively correlated with miR-22-3p in LIHC and their expression in LIHC and control tissues.

lncRNA	miRNA	Cor	*p* value	logFC	Diff *p* value
SNHG16	hsa-miR-22-3p	−0.3967	2.14E-15	0.3735	2.89E-06
GUSBP11	hsa-miR-22-3p	−0.3698	1.95E-13	0.1955	1.50E-22
FGD5-AS1	hsa-miR-22-3p	−0.3177	4.03E-10	0.5105	1.13E-09
TTC28-AS1	hsa-miR-22-3p	−0.2895	1.41E-08	0.2642	1.82E-11
LINC00630	hsa-miR-22-3p	−0.2842	2.65E-08	0.1013	4.06E-09
CD27-AS1	hsa-miR-22-3p	−0.2450	1.84E-06	0.5535	1.89E-17
LINC02381	hsa-miR-22-3p	−0.2351	4.87E-06	0.3756	0.9936
H19	hsa-miR-22-3p	−0.2198	1.99E-05	−1.4487	2.16E-07
LINC00997	hsa-miR-22-3p	−0.2174	2.47E-05	0.4144	8.01E-16

**TABLE 4:** lncRNAs are positively correlated with CCNA2 in LIHC.

lncRNA	Gene	Cor	*p* value
CD27-AS1	CCNA2	0.278336	5.94E-08
LINC00997	CCNA2	0.224875	1.33E-05
FGD5-AS1	CCNA2	0.428225	0
GUSBP11	CCNA2	0.311936	1.06E-09
SNHG16	CCNA2	0.300747	4.28E-09
LINC00630	CCNA2	0.431591	3.21E-18
TTC28-AS1	CCNA2	0.195921	0.000153

### Immune Infiltration Analysis

**FIGURE 6:**
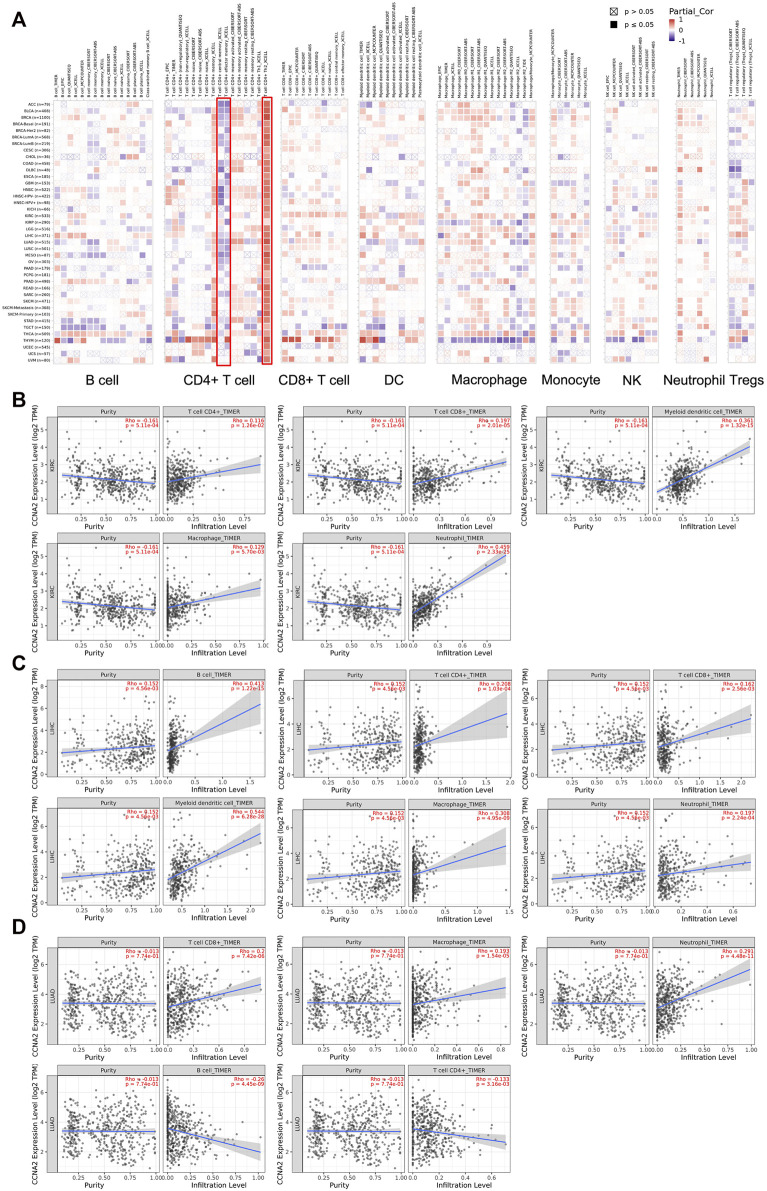
**(A)** Potential correlation between the CCNA2 expression and immune cell infiltration in all TCGA cancer types. **(B–D)** The scatter plots of related cancers generated by the TIMER algorithm, indicating association between immune infiltrates and CCNA2 expression in KIRC **(B)**, LIHC **(C)** and LUAD **(D)**.

We employed TIMER2.0 to show the landscape of CCNA2 correlating with various immune infiltrates in different cancer types (Figure 6A). Overall, the correlation of the CCNA2 expression with immune infiltration level in diverse cancer types is different. However, there is a significant positive correlation between CCNA2 and Th2 cells and significant negative correlations between CCNA2 and CD4^+^ central memory and effector memory T cells by the xCell estimation algorithm in almost all cancer types. In KIRC, the timer estimation algorithm shows that CCNA2 is positively correlated with immune infiltrating levels of CD4+T cells, CD8+T cells, myeloid dendritic cells, macrophages, and neutrophils (Figure 6B). In LIHC, CCNA2 is positively correlated with immune infiltrating levels of B cells, CD4+T cells, CD8+T cells, myeloid dendritic cells, macrophages, and neutrophils (Figure 6C). In LUAD, CCNA2 is positively correlated with CD8+T cells, macrophages, and neutrophils and negatively correlated with B cells and CD4+T cells (Figure 6D).

### Correlation Between CCNA2 and Immune Cell Biomarkers

As is shown in Table 5, CCNA2 is positively correlated with the biomarker expressions of B cells, CD4+T cells, CD8+T cells, dendritic cells, M2 macrophages, and neutrophils in KIRC. In LIHC, CCNA2 is positively correlated with the biomarker expressions of B cells, CD4+T cells, CD8+T cells, dendritic cells, and neutrophils. In LUAD, CCNA2 is positively correlated with the biomarker expressions of CD8+T cells and negatively correlated with parts of the biomarker expressions of dendritic cells and neutrophils.

**TABLE 5:** Correlation between CCNA2 and the immune cell biomarker.

Immune cell	Gene	KIRC		LUAD		LIHC	
		Cor	*p* value	Cor	*p* value	Cor	*p* value
B cell	CD19	0.321783	3.18E-14	−0.06882	0.114892	0.256426	4.99E-07
B cell	CD79A	0.193091	7.14E-06	−0.0845	0.052786	0.158396	0.002123
CD8^+^ T cell	CD8A	0.412223	0	0.228159	1.33E-07	0.202235	8.47E-05
CD8^+^ T cell	CD8B	0.38827	0	0.229167	1.16E-07	0.188851	0.00024
CD4^+^ T cell	CD4	0.351867	4.57E-17	−0.12222	0.005027	0.231919	6.20E-06
M1 macrophage	NOS2	−0.04108	0.342778	0.019806	0.650292	-0.05106	0.32468
M1 macrophage	IRF5	0.344114	3.04E-16	0.062724	0.150804	0.270353	1.23E-07
M1 macrophage	PTGS2	0.071287	0.099523	0.074417	0.088186	0.057962	0.263516
M2 macrophage	CD163	0.248252	6.62E-09	0.136455	0.001722	0.053515	0.301828
M2 macrophage	VSIG4	0.284888	2.34E-11	0.05348	0.220682	0.05507	0.28798
M2 macrophage	MS4A4A	0.299823	1.82E-12	0.056271	0.197497	0.047656	0.357903
Neutrophil	CEACAM8	0.144606	0.000795	−0.39724	2.49E-21	0.135043	0.008927
Neutrophil	ITGAM	0.271919	1.90E-10	−0.08384	0.054655	0.276869	5.95E-08
Neutrophil	CCR7	0.243437	1.31E-08	−0.17092	8.35E-05	0.068005	0.189342
Dendritic cell	HLA-DPB1	0.249344	5.67E-09	−0.31376	2.34E-13	0.144356	0.005192
Dendritic cell	HLA-DQB1	0.166207	0.000115	−0.28132	6.13E-11	0.146425	0.004579
Dendritic cell	HLA-DRA	0.277397	7.95E-11	−0.21456	7.24E-07	0.155108	0.002657
Dendritic cell	HLA-DPA1	0.292105	6.95E-12	−0.23623	4.60E-08	0.127995	0.013288
Dendritic cell	CD1C	0.090674	0.03602	−0.45993	0	0.070813	0.171754
Dendritic cell	NRP1	−0.07595	0.07923	−0.01343	0.758614	0.148927	0.003926
Dendritic cell	ITGAX	0.368298	0	−0.01266	0.77198	0.282138	3.26E-08

### Correlations Between CCNA2 and Immune Checkpoints

**FIGURE 7:**
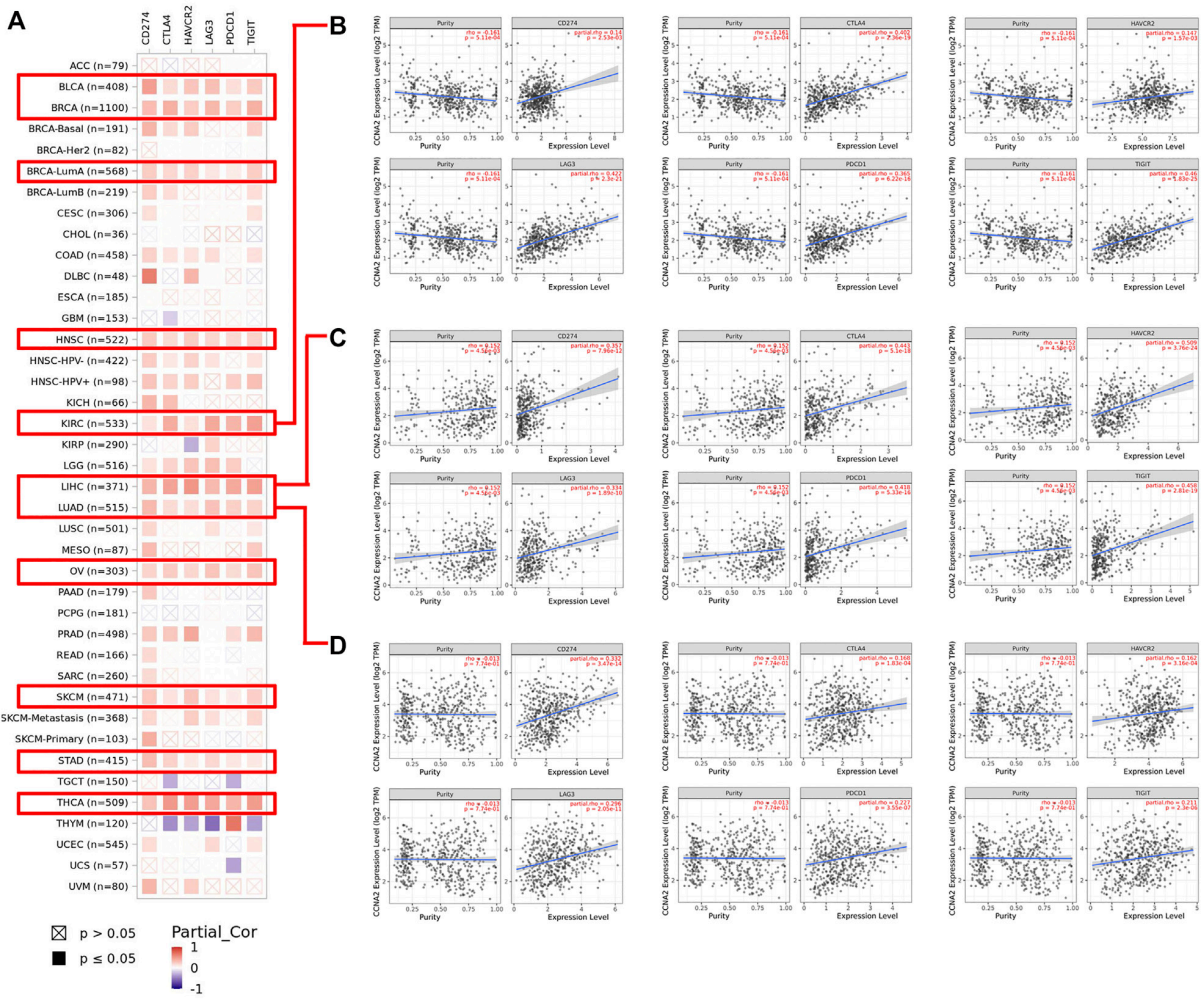
**(A)** Potential correlation between CCNA2 expression and immune checkpoints (CD274, CTLA4, HAVCR2, LAG3, PDCD1, and TIGIT) in all TCGA cancer types. **(B–D)** The scatter plots of related cancers generated by the TIMER algorithm, indicating correlation between immune checkpoints (CD274, CTLA4, HAVCR2, LAG3, PDCD1 and TIGIT) and CCNA2 expression in KIRC **(B)**, LIHC **(C)** and LUAD **(D)**.

Using the TIMER2.0 web tool, we further explored the correlations between CCNA2 and expressions of immune checkpoints (CD274, CTLA4, HAVCR2, LAG3, PDCD1, and TIGIT). Overall, CCNA2 is generally positively correlated with expressions of these immune checkpoints in most cancer types. In BLCA, BRCA, HNSC, KIRC, LIHC, LUAD, OV, SKCM, STAD, and THCA, it shows positive correlations between CCNA2 and all of the six immune checkpoints (all *p* values < 0.05, Figure 7A). However, CCNA2 is also negatively correlated with the immune checkpoints in a few cancer types. We observe a negative relationship with CTLA4, HAVCR2, LAG3, and TIGIT in THYM, CTLA4 and PDCD1 in TCGT, CTLA4 in GBM, HAVCR2 in KIRP, and PDCD1 in UCS (all *p* values < 0.05, Figure 7A). We further showed the significant positive correlations between CCNA2 and immune checkpoints in KIRC, LIHC, and LUAD (Figures 7B–D).

## Discussion

Accumulating evidence has documented CCNA2 is an important differentially expressed gene (DEG, mainly overexpressed) in various cancer types compared to normal tissues, such as LIHC (Li et al., 2021a), human epidermal growth factor 2 (HER2)+ breast cancer (Weng et al., 2021), LUSC (Gao et al., 2020), COAD (Li et al., 2021b), PRAD (Feng et al., 2021), HNSC (Zhang et al., 2020), KIRC (Zhan et al., 2021), THCA (Li et al., 2020b), medulloblastoma (Guo et al., 2020a), gastric cancer (Ji et al., 2021; Lu et al., 2021), and mantle cell lymphoma (Guo et al., 2020b). These bioinformatic results are credible because they are not only from the TCGA dataset (Li et al., 2020a; Gao et al., 2020; Zhang et al., 2020; Li et al., 2021a; Li et al., 2021b; Feng et al., 2021; Ji et al., 2021; Weng et al., 2021) but also from Gene Expression Omnibus (GEO) datasets or series (Guo et al., 2020a; Li et al., 2020a; Guo et al., 2020b; Li et al., 2020b; Gao et al., 2020; Li et al., 2021a; Li et al., 2021b; Lu et al., 2021; Weng et al., 2021; Zhan et al., 2021). However, it is unable to retrieve any available reports about a pan-cancer analysis of the CCNA2 expression across all cancer types. The results of our analysis corroborate that CCNA2 is overexpressed in all 18 cancer types with enough normal tissues from the TCGA project. When we added normal tissues from the GTEx program to other tumor types, they are still overexpressed in most cancer types. CCNA2 expression is only decreased in LAML. However, we found the K562 cell line was used as matched normal data from the GTEx database to be compared with acute myeloid leukemia (AML) cells in the TCGA database. K562 cells are from a chronic myeloid leukemia patient in a blast crisis, and they are not able to properly represent AML. Normal immature hematopoietic cells in the bone marrow (myeloblast, promyeloytes etc.) from healthy donors are a better choice. This finding suggests that the application of control tissues from GTEx has limitations. CCNA2 may not be downregulated in LAML when we choose suitable controls. Taken together, we verified that the upregulation of CCNA was widespread in most cancer types.

Furthermore, CCNA2 is thought to be a core gene in many cancer types. After getting DEGs in tumor tissues compared with normal tissues, network-based analyses (such as protein–protein interaction networks of the DEGs) verified that CCNA2 is a hub gene in LIHC (Li et al., 2021a), LUAD (Zeng et al., 2020), LUSC (Gao et al., 2020), STAD (Ji et al., 2021), PRAD (Feng et al., 2021), HNSC (Zhang et al., 2020), THCA (Li et al., 2020b), medulloblastoma (Guo et al., 2020a), mantle cell lymphoma (Guo et al., 2020b), and colorectal cancer (Wang et al., 2020). CCNA2 interacts with many other differentially expressed proteins, indicating its essential role in many cancers. In addition, we also provided substantial evidence in support of the prognostic values of CCNA2, and some of these findings were consistent with other studies. High expression of CCNA2 was connected with the poor OS in LIHC (Li et al., 2021a) and PRAD (Feng et al., 2021). CCNA2 expression was an essential component of the prognostic model in LUAD (Zhou et al., 2020), LUSC (Gao et al., 2020) and KIRC (Zhan et al., 2021).

Due to its important role in cancer, CCNA2 has become a therapeutic target for many cancer types. Chen et al. reported Roundabout homolog 1 inhibited pancreatic cancer *via* the YY1-ROBO1-CCNA2-CDK2 axis (Chen et al., 2021). The combination of metformin and pemetrexed exhibited an antiproliferative effect by affecting the cell cycle in non-small-cell lung cancer *via* downregulation of CCNA2 and CCND1, and the upregulation of CDKN1B (Wang et al., 2021b). Interestingly, CCNA2 deletion in oncogene-transformed mouse embryonic fibroblasts suppressed liver tumor formation, indicating its important role in tumorigenesis (Gopinathan et al., 2014). Although CCNA2 is related to poor prognosis and tumor-promoting function in most tumor types, COAD is an exception. The CCNA2 expression is upregulated in COAD tissues, but its high expression is related to better OS. Guo et al. (Guo et al., 2021) reported that CCNA2 deficiency in colonic epithelial cells led to epithelial changes in the mucosa, inducing inflammation and increasing cell proliferation and dysplasia in the colon. These changes make them more susceptible to chemically induced colon carcinogenesis in the mice. Meanwhile, they also verified that the higher expression of CCNA2 existed in pathological stage 1 or 2 colorectal cancer than in stage 3 or 4.

In this study, we first presented evidence of the potential ceRNA network based on CCNA2 in tumors. There was no report about the function of miR-27b-3p in KIRC. Through expression analysis, survival analysis, and correlation analysis, we found the miR-27b-3p/CCNA2 axis in KIRC. Furthermore, LINC00997 might be the regulatory lncRNA of the miR-27b-3p/CCNA2 axis in KIRC. MicroRNA-22-3p played a role in reducing tumor progression in LIHC. A study confirmed that the miR-22-3p overexpression could impair cell mobility and invasiveness in LIHC (Zhang et al., 2018b). In addition, catalpol had antitumor effects by upregulating miR-22-3p expression and targeting the metastasis associated with 1 family member 3 in LIHC (Zhao et al., 2019). Consistent with these studies, we found high expression of miR-22-3p was correlated with better OS in LIHC. CCNA2 should be one target of miR-22-3p, and SNHG16, GUSBP11, FGD5-AS1, LINC00630, CD27-AS1, and LINC00997 might be the regulatory lncRNAs of the miR-22-3p/CCNA2 axis. Among the miR-22-3p-bound lncRNAs, SNHG16 was verified as a miRNA sponge (ceRNA) and promoted LIHC metastasis and EMT progression in several studies (Li et al., 2020c; Hu et al., 2020) (not through CCNA2), and there was no report about other lncRNAs in LIHC.

We found CCNA2 played a significant but contradictory role in immune infiltration in different cancer types. For example, CCNA2 showed a significant positive correlation between CCNA2 and B cells in LIHC but a significant negative correlation in TGCT. Importantly, our work showed that there was a significant positive correlation between CCNA2 and Th2 cells by the xCell estimation algorithm. Th2 cells were thought to be associated with aggressive tumors through the production of the immunosuppressive cytokine IL-10 or through the activation of B cells. However, TH2 cells were also associated with favorable prognosis in follicular lymphoma, Hodgkin’s lymphoma, and breast cancer, which suggested a protective effect (Fridman et al., 2012). Besides, as there was a close correlation between immune checkpoints and immunotherapy, we analyzed the relationship between CCNA2 and immune checkpoints. CCNA2 was positively correlated with expressions of CD274, CTLA4, HAVCR2, LAG3, PDCD1, and TIGIT in most cancer types, which indicated the CCNA2 expression may act as a marker after immunotherapy.

Taken together, our first pan-cancer analysis of CCNA2 indicated its overexpression is widespread in different cancer types. In addition, high expression of CCNA2 is related to poor prognosis and advanced pathological stages in most cases. Through expression analysis, survival analysis, and correlation analysis, we built the upstream regulatory networks of CCNA2 in different cancer types (LINC00997/miR-27b-3p/CCNA2 ceRNA network in KIRC; SNHG16, GUSBP11, FGD5-AS1, LINC00630, CD27-AS1, and LINC00997/miR-22-3p/CCNA2 ceRNA network, miR-29b-3p/CCNA2, miR-29c-3p/CCNA2, and miR-204-5p/CCNA2 networks in LIHC; and miR-218-5p/CCNA2 and miR-204-5p/CCNA2 networks in LUAD). The CCNA2 expression is positively correlated with Th2 cell infiltration and negatively correlated with CD4^+^ central memory and effector memory T-cell infiltration. Furthermore, CCNA2 is positively associated with expressions of immune checkpoints in most cancer types. Our work of CCNA2 pan-analysis contributes to understanding the prognostic and immunological roles and potential upstream molecular mechanisms of CCNA2 in different cancers.

## Data Availability

The datasets presented in this study can be found in online repositories. The names of the repository/repositories and accession number(s) can be found in the article/Supplementary Material.
