# Supplementary figures and images for: The Predictive Competing Endogenous RNA Regulatory Networks and Potential Prognostic and Immunological Roles of Cyclin A2 in Pan-Cancer Analysis
Source: Front Mol Biosci. 2022 Apr 11;9:809509. doi: 10.3389/fmolb.2022.809509 (PMC9035520; doi:10.3389/fmolb.2022.809509)

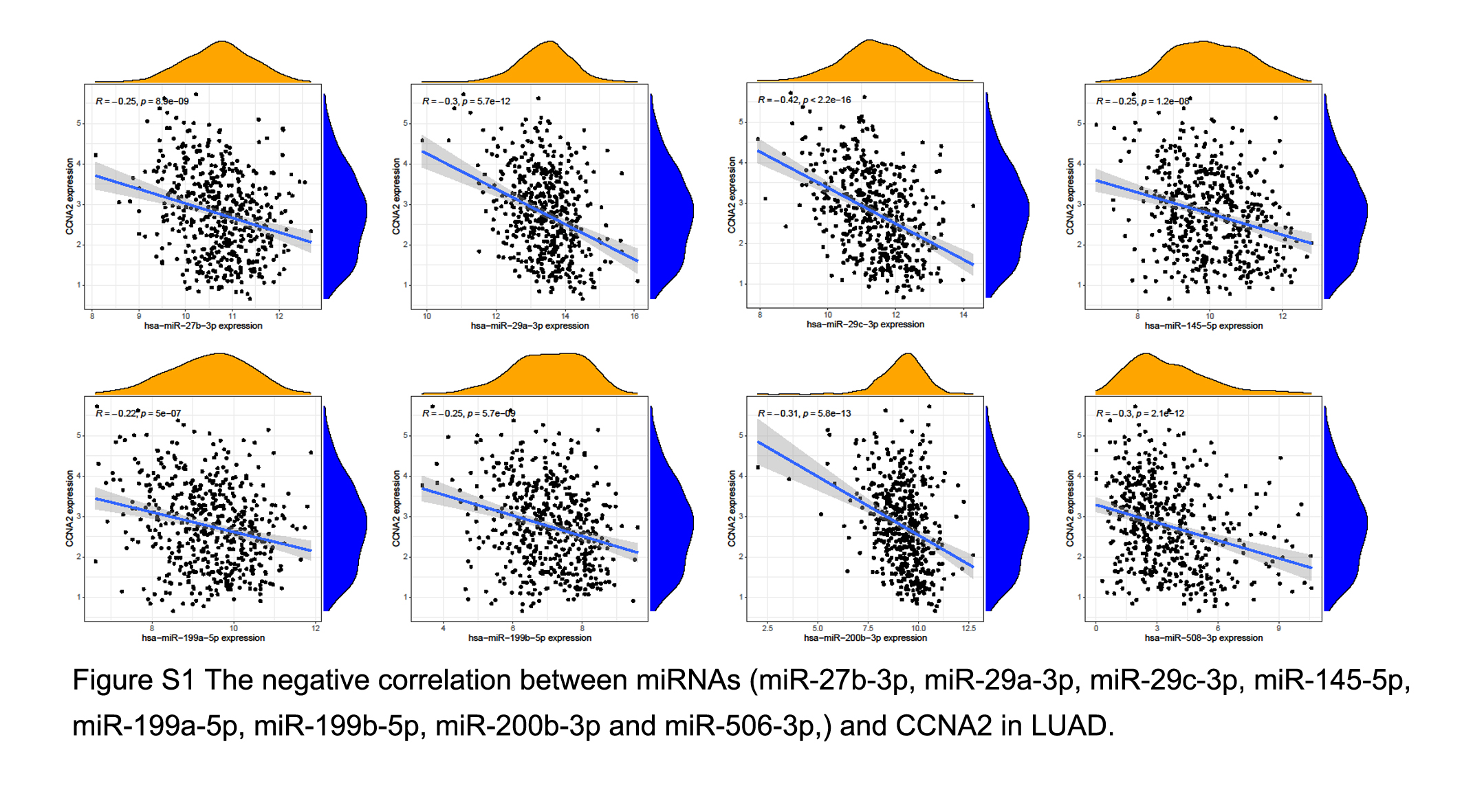

Supplement: Supplementary file 1 [file Image1.JPEG]
